# General perceptual-cognitive abilities: Age and position in soccer

**DOI:** 10.1371/journal.pone.0202627

**Published:** 2018-08-23

**Authors:** Nils Schumacher, Mike Schmidt, Kai Wellmann, Klaus-Michael Braumann

**Affiliations:** University of Hamburg, Faculty of Psychology and Human Movement, Institute of Human Movement Science, Department Sports and Exercise Medicine, Hamburg, Germany; Berner Fachhochschule, SWITZERLAND

## Abstract

Various studies suggest the importance of sport-specific cognitive and perceptual abilities in soccer. However, the role of general perceptual-cognitive abilities and the relation of age respective to position have not been clarified for soccer in detail. Therefore, it was the objective of the present study to determine the relation of age and position to general perceptual-cognitive abilities. 178 highly talented male soccer players (mean age 16.2, age range 10 to 33 years) were involved. The participants performed computer-based sustained attention and anticipation (using Vienna Test System) tests. 139 subjects (mean age 16.6) took part in visual and acoustic reaction tests (using Talent Diagnostic System). The soccer players, subdivided into age and position groups, were recruited from a youth academy of a professional soccer club and played at the highest and 2^nd^ highest national soccer competition for their age. Group differences were tested using analysis of variance. Correlations were analyzed for age and abilities.

Significant correlations and group differences were found for age and sustained attention tasks. Significant differences for position groups were observed with regard to acoustic reaction time (ART). Further, we found statistical tendencies for group differences regarding the visual reaction time (VRT), indicating that midfielders outperform defenders and strikers in simple reaction tasks. Improved skills in sustained attention tasks resulted for defenders, who worked faster and more precisely in figural tasks. Regarding general anticipation tasks differences were not found. No group differences were found in basic anticipation tasks. Our study indicates that additional research is needed to further clarify the development of general perceptual-cognitive abilities and position-specific differences in the above abilities of highly talented soccer players.

## Introduction

In team sports, especially in soccer, players continuously experience rapidly changing situations. Expert performance in this field means to perceive the situation, process the stimuli, make as fast as possible the correct decision and carry out the action at the precise moment [1,2]. Hence, expert performance involves a combination of motor and perceptual-cognitive skills [3]. The perceptual-cognitive skill in turn is defined as the ability of an individual to locate, identify and process environmental information to integrate it with existing knowledge and current motor capabilities in order to select and execute appropriate actions [4].

Recent evidence supports the so-called *Expert-performance approach* [5], which clarifies the importance of perceptual-cognitive functions in high performance sports [6–13]. Particularly, abilities such as to anticipate and make appropriate decisions occur to be important on the expert level [14,15]. Research showed that experts in team sports, e.g. in soccer or field-hockey, perform better in sport-specific tasks than novices [9–12]. For example, it was illustrated that experts are predominant regarding the ability to recognize information through opponents´ postural cues to anticipate forthcoming movements [12]. They also have superior abilities to more precisely foresee the movements of teammates and opponents [10,11]. Furthermore, there is evidence that experts have a functionally organized memory structure which leads to a reaction time and perceptual advantage in tactical decision-making [13]. Nevertheless, these sport-specific perceptual-cognitive skills may be based on enhanced fundamental abilities such as general attention, anticipation or reaction abilities [16–18].

In psychology, fundamental abilities which control behavior are summarized as executive functions (EF; also called executive control or cognitive control) [19–21]. EF`s are subdivided into core executive functions (attentional control, inhibition, working memory, cognitive flexibility) and higher-order executive functions such as reasoning, problem solving, and planning [22]. It might be useful to consider EF´s to compare cognitive abilities in different sports. Measurements of “general” EF´s provide additional data to sport-specific perceptual-cognitive tests.

The *Cognitive component skill approach* examines the relationship between sports expertise and performance of non-sport-specific, general cognition [23]. Voss et al. [17] reviewed studies which used the *Cognitive component skill approach* to examine the relationship between sports expertise and general cognitive skills. They found that elite athletes performed better in cognitive measures, with the largest effect size seen for processing speed and attention tasks. Similar results have been obtained in recent studies, which showed that elite athletes partially perform better in sustained attention tasks [18,24,25], working memory [16], inhibitory control and cognitive flexibility [16,26]. Nonetheless, research based on the *Cognitive component skill approach* yielded diverse results [27,28]. Although studies investigated the development of cognitive skills in soccer using sport-specific tasks [11,29,30], relatively few studies used the *Cognitive component skill* approach examining how general perceptual-cognitive abilities improve with age in soccer [24,31].

In neuropsychology, earlier research about brain maturation and sustained attention revealed, that development of sustained attention abilities followed a U-shaped trend, showing, that there is a significant increase from childhood to early adulthood (12-15/16 years) and a decline from young adulthood (20–30 years) to older adulthood (60–70 years) [32]. Further, there is an improvement in sustained attention skills from 8 to 16 years, but with the magnitude of gains reducing from around 10 to 11 years [33–35]. This is seen by Betts et al. [35] who observed a rapid development from the age of 5/6 years to the age of 8/9 years and a development plateau from the age of 8/9 years to the age of 11/12 years.

Approaches containing sport-specific tasks pointed out how abilities such as anticipation improve with age and experience and how highly developed these abilities are when compared to less skilled athletes [11,30]. Using the Temporal occlusion paradigm a lack of skill based differences in ability to extract task relevant information, such as postural cues [30] was found. Nevertheless, it was also claimed that tests of anticipatory performance in 11 vs. 11 player simulations revealed differences across skill groups of soccer players between 9 and 17 years. Also, age is a strong predictor of structured memory skill, anticipation in 3 vs. 3 situations and the ability to recognize situational probabilities in game patterns. Tests of visual function did not discriminate between age groups until early adulthood and it was shown that peripheral awareness improved up to the age of 13 years [11]. Results of unspecific computer-based procedures showed that highly talented adolescent (8–16 years) soccer players outperformed amateur players in inhibiting an ongoing motor response and had superior ability to attain and maintain an alert state in attention tasks [24]. In addition, general perceptual motor skills, such as simple reaction and peripheral awareness seemed to improve with age [11,31].

Summarizing, few research was concerned with the development of general perceptual-cognitive abilities of highly talented soccer players. Furthermore, it is still not clarified how young elite soccer players perform in EF´s as compared to the norm population. Therefore, research on the development of core EF´s, such as sustained attention, of highly talented soccer players could provide relevant practical applications. This could involve methodological training advices due to the knowledge about attention phases in the age classes. On the other hand, EF´s might be comparable over sports and with the general population since they are standardized.

In order to clarify the mechanisms of underlying perceptual-cognitive expertise, it is important to identify the specific mechanisms mediating expert performance within the team, such as *positional role* [36]. Earlier studies suggested that skilled defenders employed different visual search strategies, regardless of their actual playing position [37–39]. Also, Slimani et al. [40] concluded that there are differences in practical cognitive/ psychological interventions according to the playing position. Vestberg et al. [16] analyzed the link between EF´s and success in young soccer players. They showed, that results regarding working memory tasks and cognitive flexibility tasks correlate with the number of scored goals. Latter indicates already differences of general perceptual-cognitive abilities within the team. The knowledge about different position roles may provide relevant information for practitioners.

Currently the question regarding appropriate methods to measure general perceptual-cognitive skills is not conclusively clarified [41,42]. Nevertheless, computer-based systems, like the Vienna Test System (VTS), seem to provide an objective and promising approach for measuring cognitive and perceptual abilities [43,44].

Although few studies investigated the development of performance relevant-abilities in elite youth soccer, little is known about the development of general perceptual-cognitive skills (*Cognitive component skill approach*) and position-specific differences.

For talent identification, development and future research it is of advantage to better understand the development of basic cognitive functions and specific mechanisms mediating expert performance within the team, such as the *role of position*. Hence, for age dependent training methods knowledge of basic cognitive core functions might be of interest for practitioners. For example, sustained attention abilities might influence general attention phases.

In our study age groups and position groups were compared separately regarding differences in three important components: sustained attention, anticipation of time and movement and reactive behavior (visual and acoustic). We considered age ranges as defined by the Union of European Football Associations (UEFA). The subdivisions include players of age under 12 years (U12), under 13 years (U13), under 14 years (U14), under 15 years (U15), under 16 years (U16), under 17 years (U17), under 19 years (U19), under 23 years (U23) as well as the professional team (PT).

In accordance to some conceptualizations concentration ability has been defined as the ability to sustain attention (i.e. speed and precision) over relatively long periods [45,46]. Following the *Cognitive component skill approach* consequently non-sport-specific measures were implemented, using the computer-based Vienna Test System (VTS) for sustained attention and anticipation measurements. The Talent Diagnostic System (TDS) was used for measuring acoustic and visual reactive behavior.

The purpose of the presented study was to investigate general perceptual-cognitive abilities of highly talented soccer players. Our first objective was to better understand the development of general perceptual-cognitive abilities (sustained attention, reaction, anticipation) between age groups (from the age of 11 to the age of 33 years).

Second objective was to analyze the position role and examine which general perceptual-cognitive abilities (see above) discriminate between soccer positions (goalkeeper, defender, midfielder and striker).

We hypothesized that sustained attention, reaction (visual and acoustic) and time and movement anticipation abilities increase with age of highly talented soccer players. Due to earlier findings regarding sustained attention we also hypothesized significant differences between the age groups of U17/U16 and the age groups of U13/U12 and a development plateau after the age of 16 years. Moreover, we hypothesized that significant position related differences concerning sustained attention, reaction (visual and acoustic) and time and movement anticipation abilities exist.

## Materials and methods

### Ethics statement

Ethical approval for the present study was obtained from the ethical commission of the faculty of social and behavioral science, Friedrich-Schiller-University Jena (protocol no. FSV 15/14). Data collection took place anonymously and all measurements were noninvasive. Further, to meet the ethical standards all participants and parents and/ or legal guardians were in detail informed about the procedure of measurements (including: a statement that the study involves research, explanation of the purpose of the research, description of the procedures, description of any reasonable foreseeable risks or discomforts, duration of the measurements, explanation whom to contact for answers about the research and the research subjects´ rights, a statement that participation is voluntary and the subject may discontinue participation at any time without penalty or loss of benefits). All participants and parents and/ or legal guardians gave their written informed consent due to participation. Only participants following these rules were included in the study.

### Participants

Highly talented male soccer players (N = 178) were recruited from a German 2^nd^ league soccer club. In addition to the entire young talent center (U12-U23), the professional team (PT) of the club also took part. Elite players played at the highest (U19, U17, U15, U13, U12), 2^nd^ highest (PT, U16, U14) and the 4^th^ highest (U23) level of national competition. The age classes used in our analysis were defined by the UEFA age classification. In addition, members of the U19 had to have reached the age of 18 at the beginning of the season, for U17 they had to have reached the age of 16 at the beginning of the season, etc. for the other teams. However, in the professional team (PT) age is not determined by the national football association. The number of recruited players for each measurement was dependent on the team size of the respective team and of the physical constitution. In terms of injury some subjects (n = 39) didn´t participate in the simple reaction test. The demographics of elite players for age and position groups are shown in Table 1. Represented are mean ages (M), standard deviations (SD) and numbers of participants (N) for each group and measurement (sustained attention & anticipation, visual & acoustic reaction). 178 highly talented soccer players (mean age 16.2, SD 4.8) took part in the measurement for focused attention and anticipation. 139 of these subjects (mean age 16.6, SD 5.0) joined the measurements of visual and acoustic reaction. Details of the game position were requested by the player or indicated by the coach.

**Table 1:** Demographics of elite players in age and position groups.

	**Sustained Attention & Anticipation**		**Visual & Acoustic Reaction**	
**Age Group**	**N**	**Age, M(SD)**	**N**	**Age, M(SD)**
**PT**	26	24.7 (3.8)	24	24.6 (3.9)
**U 23**	17	20.3 (0.6)	15	21.0 (3.9)
**U 19**	22	17.3 (0.6)	15	17.3 (0.6)
**U 17**	21	15.3 (1.1)	16	15.3 (1.8)
**U 16**	19	14.8 (0.4)	17	14.8 (4.4)
**U 15**	21	13.8 (0.5)	16	13.8 (0.6)
**U 14**	20	12.9 (0.7)	14	12.9 (0.7)
**U 13**	15	12.1 (1.1)	9	11.9 (0.3)
**U 12**	16	10.8 (0.4)	13	10.7 (0.5)
**Total**	178	16.2 (4.8)	139	16.6 (5.0)
**Position Group**				
**Goalkeeper**	20	16.8 (5.8)	13	18.5 (9.9)
**Defender**	63	16.7 (5.2)	47	17.4 (5.7)
**Midfielder**	64	16.3 (4.0)	53	16.2 (4.0)
**Striker**	31	14.7 (4.5)	26	15.1 (4.7)
**Total**	178	16.2 (4.7)	139	16.6 (5.0)

*Note*: Values shown are the mean (M) and standard deviation (SD). PT = Professional team. Represented are mean ages (M), standard deviations (SD) and numbers of participants (N) for each group and measurement (Sustained attention & Anticipation, Visual & acoustic reaction).

### Materials

#### Visual and acoustic reaction

Simple visual and acoustic reaction time was measured using Talent Diagnostic System (TDS) [47]. Therefore, the subjects had to stand on a two-part ground contact plate and executed a simple reaction due to an acoustic or a visual stimulus given by a computer. The visual target stimulus was a red square presented on a black computer screen. The acoustic stimulus was given by a simple “beep”. Simple visual and acoustic reaction times were measured separately and administered in the same order for each participant. For standardized execution subjects were requested to position each foot in the middle of one part of the two-part ground contact plate. The rear end of the plate was fixed 50 cm apart from the table on which the computer screen was located. The computer screen was installed in a distance of 80 cm of the rear end of the panel. To assess the reaction time subjects were then asked to build a knee angular of 170°, a slight body tension and to watch the display. Further, the participants were instructed to unload the plate as quickly as possible if a visual/ acoustic signal was given. Moreover, subjects were advised that the quickest way to unload the plate was to lower the buttocks, the upper body, the head and the upper extremities. The stimulus appeared 8 times in a temporal random distance of 2–4 seconds. The program calculated the average deviation of 4 mid-values (4 extreme values were discarded) for each measurement in milliseconds (ms). The entire series was repeated if an experiment was performed more than 2 times irregularly within a series (e.g. by interruption).

#### Anticipation of time and movement

Anticipation of time and movement were measured using TMA (Time and Movement Anticipation) by Schufried GmbH [48]. The participants had to imagine the effect of a movement by correctly estimating the movement of a green ball moving slowly in a white background display. For measuring the anticipation of time, at an unpredictable moment the green ball disappeared and two red lines appeared instead. One line passed through the point at which the ball had just disappeared. The other was the target line. The subjects´ task was to indicate when the ball would reach the target line. In case the participants considered the moment to be correct, they pressed a green button. In the instruction phase the respondents received feedback: Then they were shown where the dot actually was at the time they pressed the button and how its course continued. In the test phase no feedback was given. The tasks varied in difficulty. First the ball moved in a linear direction, later followed a curvilinear path.

Anticipation of time was measured by the median of the time deviations (TD) for all items in seconds. The time deviation was the difference between the anticipated time of the ball crossing the second line and the actual time the ball crossed the line. Anticipation of movement was measured by the median of the direction deviations (DD) for all items in pixels. The direction deviation was the difference between the anticipated point of the ball crossing the second line and the actual point the ball crossed the line. Reliability and validity have been reported for the overall test elsewhere [49].

#### Sustained attention

Sustained attention was assessed using the arrow subtest of Vienna Test System (VTS) [49]. Similar to the d2-R test [50], participants had to sustain attention over a relatively long period. This subtest is one of three tests of the Complex Concentration Test (CCT) [51] and lasted about 6 minutes. The test consists of four different types of arrows (figural material) randomly presented in a row and pointing in one of four directions. Subjects were instructed to respond to targets (only the simple arrows pointing to the upper right corner) by pressing a green button (on the left side). Non-targets (any other combination of arrow type and direction) had to be replied by a red button. Non-targets were used as distractors and the relation of targets (75%) to distractors (25%) was constant. The task complexity was achieved using arrows of different figural complexity and dimensional overlap. The test period was determined to 11 minutes (min), so that the number of correct responses was dependent to the pace of work and the accuracy. Therefore, subjects were instructed to respond as quickly and accurately as possible to the appeared arrows.

The number of correct responses (CR), within the 11-minute test period, indicated the pace of concentrated work. In order to determine the tendency to concentration errors (E), the percentage of wrong responses was calculated. Adequate validity had been reported for the overall test (CCT) [51,52].

### Procedure

The measurements consisted of overall three subtests (Visual and Acoustic Simple Reaction Test, Time and Movement Anticipation Test, Arrow Subtest) and were recorded onetime at the beginning of the season in the context of a sports medicine examination at the University of Hamburg, Department of Sports and Exercise Medicine. Measurements were carried out with the VTS [48] and TDS [47], both computer-based systems. Single sessions for each individual participant were executed using standardized instructions. The total amount of administration was about 45 minutes and each measurement was conducted in the same order in a quiet room.

### Statistical analysis

SPSS version 22.0 was used for all statistical analyses [53]. Mean difference between age group and position group for all of the six variables (number of correct responses (CR), percentage of error (E), time deviation in seconds (TD), deviation of movement in pixels (DD), visual and acoustic reaction time (VRT, ART)) were examined using separate one-way ANOVAS. The between participant factors were age group (PT, U23, U19, U17, U16, U15, U14, U13, U12) and position group (goalkeeper, defender, midfielder, striker). The Bonferroni post hoc test was utilized to compare pairwise the mean differences between age groups and separately between position groups. The Alpha level was set at 0.05. Effect size was calculated by Eta square (η^2^). According to Cohen´s boundaries [54] the effect size of the one-way ANOVA was set to .01 (small effect), .06 (moderate effect) and .14 (large effect).

Correlations between age groups and the variables CR, E, TD, DD, VRT, ART was investigated using Spearman´s rho. Alpha was set at .05 and effect sizes were calculated in terms of Correlation Coefficient (r) with values .10, .30, .50 referring to small, moderate and large correlations.

## Results

### Relation between age and abilities

Descriptive statistics and Analysis of Variance (ANOVA) for variables and age groups are shown in Table 2. Significant differences were found between age groups and CR (F(8,169) = 9.5, p < .001, η^2^ = .45) and between age groups and percentage of errors (E: F(8,169) = 2.45, p = .015, η^2^ = .12). A large correlation was found between age and correct responses (r(176) = .56, p = .001). The correlation between age and E revealed (r(176) = -.23, p = .002) a small effect. Results for each variable are described in detail below.

**Table 2:** Descriptives and ANOVA for age groups referring to sustained attention, anticipation and visual & acoustic reaction.

	Sustained Attention		Anticipation		Visual & Acoustic Reaction	
Age Group	CR [N]	E [%]	TD [s]	DD [Pixel]	VRT [ms]	ART [ms]
	M (SD) CI95%	M (SD) CI95%	M (SD) CI95%	M (SD) CI95%	M (SD) CI95%	M (SD) CI95%
**PT**	1650.7 (206.6) [1567.3–1734.1]	3.02 (2.11) [2.17–3.87]	1.09 (.73) [.79–1.38]	55.27 (23.27) [45.87–64.67]	308.54 (24.7) [298.09–318.99]	266.21 (20.94) [257.37–275.05]
**U 23**	1520.9 (218.4) [1408.6–1633.2]	4.94 (3.54) [3.12–6.76]	1.10 (.46) [.86–1.33]	52.88 (15.03) [45.15–60.61]	318.67 (59.48) [285.73–351.61]	282.33 (42.94) [258.56–306.11]
**U 19**	1542.6 (174.2) [1465.4–1619.8]	5.57 (3.22) [4.14–7.00]	1.25 (.67) [.96–1.55]	56.95 (23.16) [46.69–67.22]	315.80 (26.47) [301.14–330.46]	277.53 (27.76) [262.16–292.91]
**U 17**	1525.1 (186.2) [1440.3–1609.8]	5.30 (3.58) [3.67–6.93]	.98 (.66) [.68–1.28]	69.67 (32.97) [54.66–84.68]	303.06 (35.13) [284.34–321.78]	259.56 (29.87) [243.64–275.48]
**U 16**	1491.6 (247.5) [1372.3–1610.9]	5.46 (2.78) [4.30–6.98]	.99 (.52) [.75–1.24]	60.11 (17.93) [51.46–68.75]	332.82(44.52) [309.93–355.71]	280.12 (35.85) [261.68–298.55]
**U 15**	1404.3 (203.1) [1311.8–1496.7]	6.00 (4.15) [4.11–7.89]	1.02 (.52) [.78–1.25]	66.29 (24.33) [55.21–77.36]	325.69 (33.90) [307.63–343.75]	284.75 (44.32) [261.13–308.37]
**U 14**	1341.0 (201.8) [1246.6–1435.4]	4.99 (2.40) [3.87–6.11]	1.01 (.53) [.76–1.26]	55.40 (21.06) [45.54–65.26]	351.71.(54.06) [320.50–382.93]	293.29 (41.08) [269.57–317.00]
**U 13**	1246.3 (265.3) [1094.9–1397.6]	7.73 (5.78) [4.53–10.93]	.84 (.41) [.61–1.07]	62.53 (22.41) [50.12–73.21]	343.56 (42.52) [310.87–376.24]	268.67 (51.35) [229.19–308.14]
**U 12**	1211.4 (158.7) [1126.8–1295.9]	5.49 (3.74) [3.49–7.48]	1.03 (.44) [.79–1.27]	62.63 (19.86) [52.04–73.21]	351.08 (129.92) [272.56–429.59]	268.00 (46.12) [240.13–295.87]
**ANOVA**	F(8,169) = 9.5, p < .001	F(8,169) = 2.45, p = .015	F(8,169) = .70, p = .69	F(8,169) = 1.2, p = .30	F(8,130) = 1.60, p = .13	F(8,130) = 1.29, p = .25
**Effect**	η^2^ = .45	η^2^ = .12	η^2^ = .03	η^2^ = .06	η^2^ = .10	η^2^ = .08

*Note*: Values shown are the mean (M), standard deviation (SD) and 95% Confidence Interval (CI95%). Results of Analysis of Variance (F) are presented with P values and degrees of freedom. Effect size is shown by eta-square (η^2^). PT = Professional team. CR = Correct responses (in numbers) in measurement of sustained attention. E = Percentage of errors in measurement of sustained attention. TD = Time deviation in the anticipation test measured in seconds [s]. DD = Direction deviation in the anticipation test measured in [pixel]. VRT = Visual reaction measured in milliseconds [ms]. ART = Acoustic reaction measured in milliseconds [ms].

#### Sustained attention

**Fig 1:**
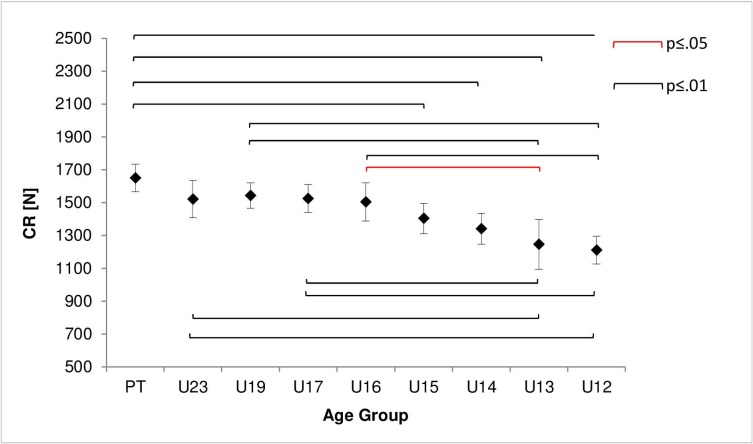
Age Group differences for correct responses (CR). Shown are means and 95% confidence interval (CI95%) (error-bars). Square brackets indicate significant differences between groups at alpha level .05 (red) and .01 (black).

One-way ANOVA demonstrated significant differences between age groups for CR (F(8,169) = 9.5, p < .001, η^2^ = .45) and E (F(8,169) = 2.45, p = .015, η^2^ = .12). As shown in Fig 1, for CR post hoc analyses indicated significant differences to U12 and U13 for all groups except U14 and U15. We observed differences to other groups only for PT (PT vs. U14 (309.69, 95%-CI [108.07, 511.31], p < .001), PT vs. U15 (246.41, 95%-CI [47.52, 445.30], p = .003)). Significant group differences for CR are shown in detail in Fig 1.

**Fig 2:**
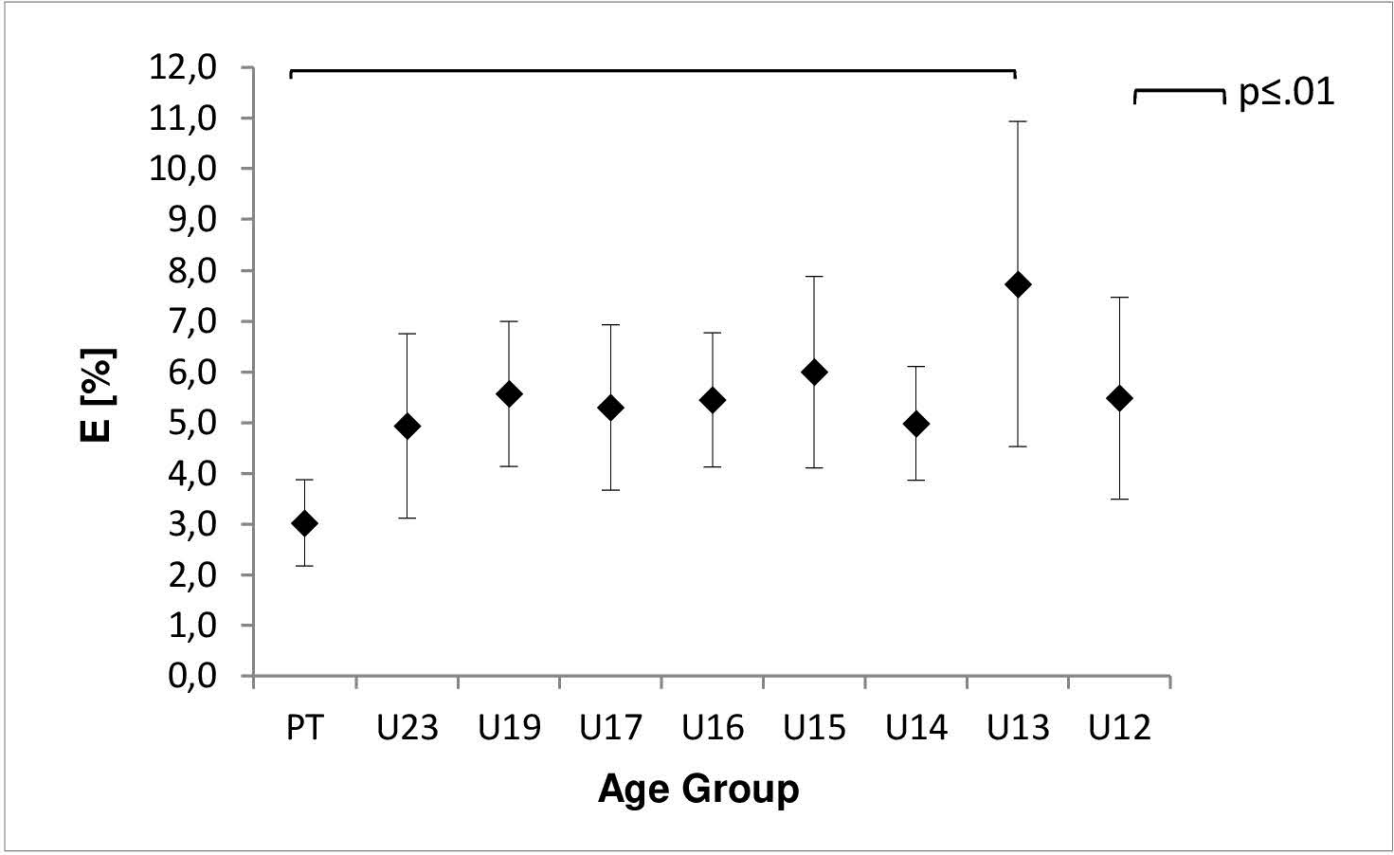
Percentage of error (E) dependent on age group. Shown are the mean and 95% confidence interval (error-bars). Square brackets indicate significant differences between groups at alpha level .01 (black).

For percentage of errors significant group differences were found (E: F(8,169) = 2.45, p = .015, η^2^ = .12) and, according to Cohen´s boundaries, a large effect size was achieved. Fig 2 shows mean differences for groups in E. Post hoc analysis revealed a significant difference between PT and U13 (-4.71, 95%-CI [-8.40, -1.02], p = .002).

The correlation between age and CR (r(176) = .56, p = .001) referred to a large effect size. On the other hand the correlation between age and E revealed (r(176) = .23, p = .002) a small effect.

#### Anticipation

No significant differences between age groups were observed for the time deviation (TD: F(8,169) = .70, p = .69) and direction deviation (DD: F(8,169) = 1.2, p = .30). However, effect size indicated a small effect for TD (η^2^ = .03) and a medium effect for DD (η^2^ = .06). No significant correlation was found between DD and age (r(176) = -.07, p = .36) and between TD and age (r(176) = -.15, p = .06).

#### Visual & acoustic reaction

ANOVA showed no significant differences between age groups for visual (VRT: F(8,130) = 1.60, p = .13) and acoustic reaction time (ART: F(8,130) = 1.29, p = .25). Eta Square indicated a large effect for VRT (η^2^ = .10) and ART (η^2^ = .08). Negative correlation with a small effect size was found between age and VRT (r(137) = -.27, p = .001). No significant correlation was revealed between age and ART (r(137) = -.06, p = .49).

### Relation between position and abilities

Descriptives and ANOVA for variables and position groups are shown in Table 3.

**Table 3:** Descriptives and ANOVA for position groups referring to sustained attention, anticipation and acoustic & visual reaction.

	Sustained Attention		Anticipation		Visual & Acoustic Reaction	
Position Group	CR [N]	E [%]	TD [s]	DD [Pixel]	VRT [ms]	ART [ms]
	M (SD) CI95%	M (SD) CI95%	M (SD) CI95%	M (SD) CI95%	M (SD) CI95%	M (SD) CI95%
**Goalkeeper**	1494.0 (216.1) [1392.8–1595.1]	4.81 (4.05) [2.91–6.70]	1.06 (.47) [.84–1.28]	58.05 (26.54) [45.63–70.47]]	321.69 (39.14) [298.04–345.34]	273.23 (28.44) [256.05–290.42]
**Defender**	1498.7 (259.9) [1433.3–1564.2]	4.67 (3.62) [3.76–5.58]	.98 (.52) [.84–1.28]	59.22 (24.50) [53.05–65.39]	324.15 (37.41) [313.17–335.13]	287.85 (35.44) [277.45–298.26]
**Midfielder**	1445.3 (246.5) [1383.8–1506.9]	5.44 (3.59) [4.55–6.34]	1.12 (.69) [.95–1.29]	61.83 (21.23) [56.52–67.13]	314.94 (48.49) [301.58–328.31]	260.81 (35.81) [250.94–270.68]
**Striker**	1367.9 (212.9) [1289.9–1446.0]	6.34 (3.20) [5.16–7.51]	1.03 (.45) [.87–1.20]	58.94 (22.51) [50.68–67.19]	350.42 (91.13) [313.61–387.23]	283.15 (39.26) [267.30–299.01]
**ANOVA**	F(3,174) = 2.21, p = .09	F(3,174) = 1.66, p = .18	F(3,174) = .63, p = .59	F(3,174) = .22, p = .88	F(3,135) = 2.44, p = .07	F(3,135) = 5.26, p = .002
**Effect**	η^2^ = .04	η^2^ = .03	η^2^ = .01	η^2^ = .003	η^2^ = .05	η^2^ = .12

*Note*: Values shown are the mean (M), standard deviation (SD) and 95% Confidence Interval (CI95%). Results of Analysis of Variance (F) are presented with P values and degrees of freedom. Effect size is shown by eta-square (η^2^). PT = Professional team. CR = Correct responses (in numbers) in measurement of sustained attention. E = Percentage of errors in measurement of sustained attention. TD = Time deviation in the anticipation test measured in seconds [s]. DD = Direction deviation in the anticipation test measured in [pixel]. VRT = Visual reaction measured in milliseconds [ms]. ART = Acoustic reaction measured in milliseconds [ms].

ANOVA revealed significant group difference in acoustic reaction time (ART: F(3,135) = 5.26, p = .002, η^2^ = .12), indicating that midfielders had significant faster acoustic reactions than defenders (-27.03ms, 95%-CI [-46.23, -7.85], p = .002). Statistic tendency was observed for group differences in visual reaction time (VRT: F(3,135) = 2.44, p = .07, η^2^ = .05), illustrating that midfielders had significant faster visual reactions than strikers (-35.48ms, 95%-CI [-70.92, -.03], p = .002). Further, ANOVA revealed a tendency for group differences in CR (F(3,174) = 2.21, p = .09).

#### Sustained attention

**Fig 3:**
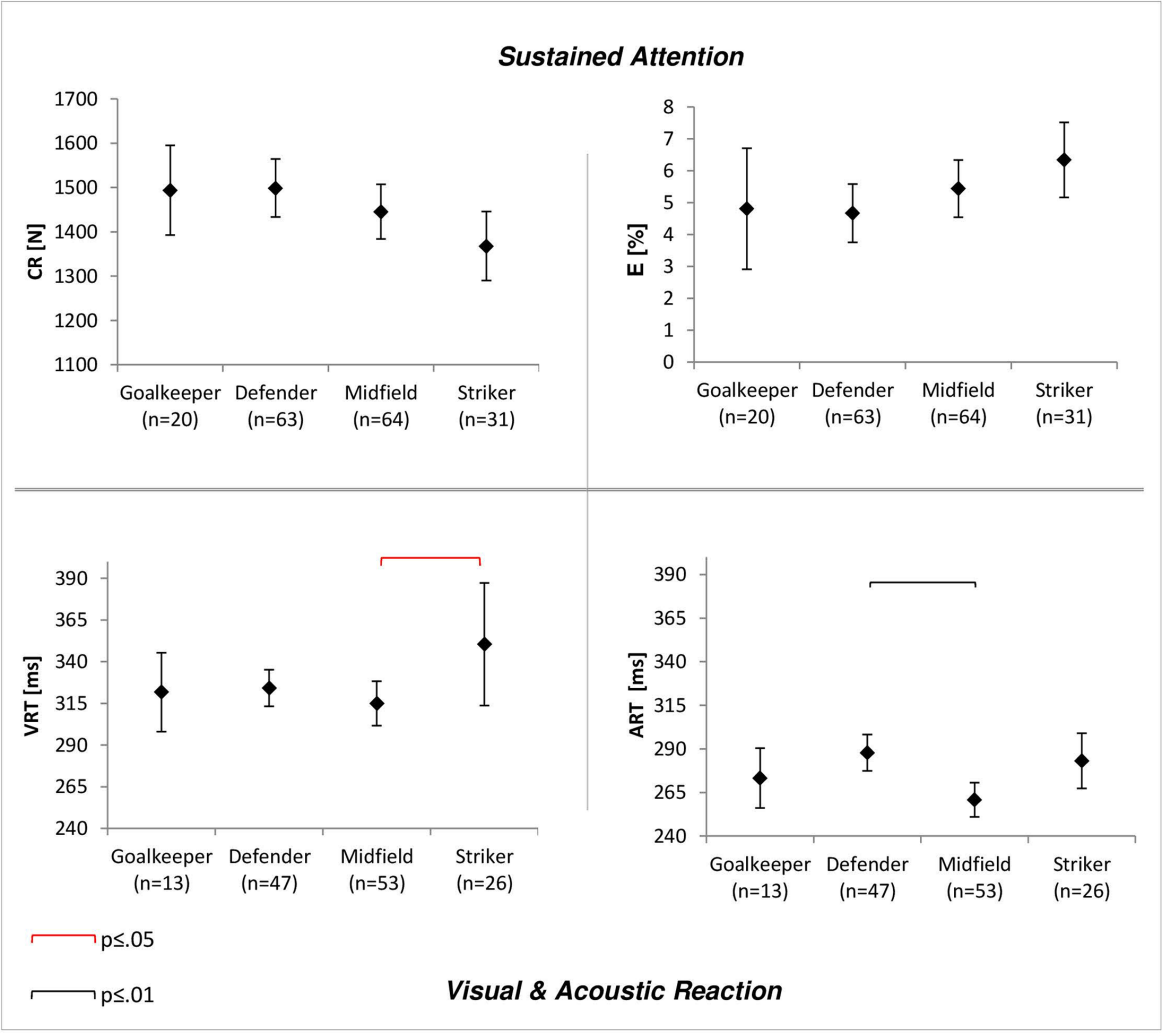
Differences between position groups for sustained Attention and reactive behavior. Shown are means and 95% confidence interval (error-bars). Square brackets indicate significant differences between groups at alpha level .05 (red) and .01 (black). CR = Correct responses. E = Percentage of errors. VRT = Visual reaction time. ART = Acoustic reaction time.

ANOVA results for CR and E are shown in Fig 3. Effect size indicated a small effect for group differences in CR (η^2^ = .04). However, only statistical tendency for group differences were observed (F(3,174) = 2.21, p = .09) with largest mean differences between defenders and strikers (130.83, 95%-CI [-11.38, 273.03], p = .09), meaning that defenders gave more correct answers in sustained attention tests. But these differences were not significant. For E no significant group differences were indicated (F(3,174) = 1.66, p = .18, η^2^ = .03). A small effect size was observed.

#### Anticipation

Neither in TD (F(3,174) = .63, p = .59) nor in DD (F(3,174) = .22, p = .88) group differences were found, indicating no differences in TD and DD between strikers, midfielders, defenders and goalkeepers.

#### Visual & acoustic reaction

Results for ART and VRT are illustrated in Fig 3. ANOVA showed significant group differences in ART (F(3,135) = 5.26, p = .002, η^2^ = .12) and a statistical tendency for group differences in VRT (F(3,135) = 2.44, p = .07, η^2^ = .05). Significant differences in ART were found between midfielders and defenders (-27.03ms, 95%-CI [-46.23, -7.85], p = .002). Statistical tendency for differences between midfielders and strikers (-22.35ms, 95%-CI [-45.27, .59], p = .06) was determined, meaning that midfielders had superior simple acoustic reactions comparing to defenders and strikers. For VRT significant differences between midfielders and strikers (-35.48ms, 95%-CI [-70.92, -.03], p = .002) were revealed, implying that midfielders had faster simple visual reactions compared to strikers.

## Discussion

Our study reveals that age seems to have a major impact on general perceptual-cognitive abilities of highly talented soccer players. Results referring to sustained attention are in agreement with general knowledge about brain maturation [32–35]. Furthermore, we observed a dependency between position and general perceptual-cognitive abilities in soccer. These results might support the *Cognitive component skill approach* [23] and demonstrate that further research on general perceptual-cognitive abilities in highly talented athletes should be performed.

### Age and abilities

As hypothesized, regarding age and sustained attention we found significant correlations concerning correct responses (CR) as well as the corresponding percentage of error (E). Furthermore, moderate to large effect sizes for group differences in CR and in E were detected for in sustained attention tasks.

The obtained Post-hoc test revealed significant group differences for all groups (PT, U23, U19, U17, U16), except U14 and U15, comparing to U12 and U13, for CR. Further, when compared U16 to older ages (U17, U19, PT) no significant group differences were obtained. These findings show a linkage with earlier research about brain maturation and sustained attention [32–35]. Similar to results of Betts et al. [35] and other neuropsychology research groups [32–34] we received a steady improvement in sustained attention skills from the age of 11 years till 16 years. A development plateau was found from the age of 16 years.

Also, our results in terms of sustained attention agree with results of sport-specific measurements. Previous sport-specific investigations approaches recognize age as a strong predictor of structured memory skill and the ability to recognize game patterns in sport-specific tests [11]. Similar to results of Ward [11], claiming that the greatest amount of errors in recalling structured patterns of play was between U11 and U13, our results indicate weaker ability to respond fast and precisely to prolonged figural attention tasks in these age classes. Taking into account that tests of working memory capacity and executive functions share a common underlying executive attention component and that results of working memory tasks have a strong relationship to attentional control [55,56], it might be conceivable that skills such as to recognize game patterns and to memorize structures of game scenes in soccer share common underlying attention components. This could be interpreted as the existence of a link between sport-specific skills and general cognitive abilities.

Moreover, when excluding U12 and U13, the professional team (PT) was the only team which differed significantly from other teams in CR (U15, U14). The result could be explained as follows:

Either the age has an influence even in the adulthood (players of U23 and PT are grown up, but showed differences in mean age) or the better responses (compared to the U23) are due to the expert level of the PT and the level of expertise has an impact on the ability to respond fast and precisely to prolonged figural attention tasks. Latter would be in line with studies [16,18,24], which showed differences in general cognitive abilities between expert and novice athletes. Nevertheless, significant group differences in CR were not found between U23 and PT.

The present study implies that sustained attention tasks could be utilized to differentiate between age groups in highly talented soccer players.

Although, in some cases athletes with weaker attentional abilities might develop strategies to be successful in their sports, attention abilities could be important for successful skill development in soccer. Therefore, additional research should aim at investigations which clarify the importance of sustained attention abilities relatively to soccer skills. These studies should firstly focus on comprehensive methods and diverse approaches, which measure ability to sustain and maintain attention not only with figural items. Secondly, the role of higher EF variables should be investigated, e. g. when players in their cognitive processes manipulate data through their short-term memory, working memory, cognitive flexibility or inhibition to make successful decisions.

Especially for younger players video-based sport-unspecific tasks are not as motivational as sport-specific tasks and therefore results for younger ages could underestimate sustained attention abilities. Furthermore, in future research it could also be helpful to determine physiological parameters (e.g. prefrontal blood Oxygen saturation) in order to better understand the underlying mechanisms.

For linking the *Cognitive component skill approach* and the *Expert-performance approach* we suggest, that future studies should examine the relationship between soccer-specific skills such as the skill to memorize structures of game scenes or to recognize game patterns and general attention ability.

In time and movement anticipation measurements no significant group differences were detected. Our results are contrary to recent findings about soccer-specific anticipation skills, which implied that age is a strong predictor for anticipation abilities [11]. Due to Zwierko and Baláková [27,57], demonstrating that TMA might not discriminate athletes from non-athletes and only partially (concerning movement anticipation) discriminate athletes of different expert levels in soccer, our results suggest that TMA might not be usable to discriminate between age or position groups in highly talented soccer players.

It might be hypothesized, that there are no differences in the general ability to anticipate between age groups, position groups or athletes from different skill levels. However, sport-specific anticipation tests revealed evidence for differences between age groups [11] and it was observed that this is not attributed to differences in the ability to extract task relevant postural cues [30]. Taking these results into account, additional research approaches should aim to examine differences in sport-specific anticipation measurements to appreciate underlying processes and, possibly, general abilities.

Our analysis of visual and acoustic reaction suggest no impact of age on reaction abilities. Neither any correlations, nor any group differences were found. These findings are contrary to results shown by Vänttinen et al. [31], indicating an improvement of simple reaction abilities with age. These age-based differences could be attributed to the complexity of tasks. As concluded in former studies the effect of age being more marked for complex reaction tasks [47,48] and therefore our visual and acoustic reaction tasks possibly might not be as complex enough.

However, the present results showed no impact of age on acoustic and visual reaction times. For practitioners these findings might indicate that reaction training could be effective for younger age classes.

### Position and abilities

The second goal of our study was to analyze the position role and examine which general perceptual-cognitive abilities discriminate between soccer positions (goalkeeper, defender, midfield and striker). It was our hypothesis that significant position related differences in sustained attention, reaction (visual and acoustic) and time and movement anticipation abilities exist.

Our findings revealed significant group differences in acoustic reaction time (ART) and statistical tendency for group differences in correct responses (CR) and visual reaction time (VRT). Post hoc analysis showed a statistical tendency for group differences between defenders and strikers, indicating that defenders work faster and more precisely than strikers in prolonged figural attention tasks. Although these results show only a statistical tendency for group differences in CR and no significant difference in E, our results portend that defenders outperform strikers in figural sustained attention tasks. Such position-specific differences are discussed by Vestberg et al. [16] and Wylie et al. [58]. Vestberg et al. [16] showed that results of working memory tasks and cognitive flexibility tasks relate to the number of scored goals during the season. Wylie et al. [58] investigated collegiate football players and identified differences between positions in cognitive control.

In addition, this could also be an indication for different position-specific behavioral patterns. An explanation could be that back position players (e.g. goalkeeper, defender) generally work more precisely, faster and rather avoid mistakes than front position players (e.g. midfielder, striker). However, the above interpretation is vague and further studies are definitely needed to examine the differences in behavioral patterns between the playing positions.

What is more, differences in visual and acoustic reactions between playing positions indicate that midfielders reached significant superior visual reaction times compared to strikers and showed significantly better acoustic reactions than defenders. Moreover, a statistical tendency for group differences between midfielders and strikers in ART, suggest that midfielders react faster in acoustic reaction tasks. These findings imply that midfielders outperform their teammates, especially strikers, in visual and acoustic reaction tasks and have superior abilities in simple reaction tasks. Although this explanation needs further evidence, the present results could be related to differences in practical cognitive/ psychological interventions according to playing positions [40].

Accordingly, as described above, the age might have an impact on general perceptual-cognitive abilities in highly talented soccer players. Therefore, our results (independent of age) might underestimate the difference between position groups and future research should be directed to examine age related differences for position groups. Inversely to studies, supporting that the position role has an influence on visual search behavior [37–39], which seems to be associated with improved sport-specific ability to foresee game relevant events, we couldn´t observe any group differences between time/ movement anticipation and position groups.

It should be mentioned that sample sizes for strikers and goalkeepers were much smaller than for defenders and midfielders. Due to this bias there might be an underestimation regarding the observed mean differences in sustained attention tasks and especially in visual and acoustic reaction tasks. Investigations with larger sample sizes for each position, primarily for goalkeeper and striker groups are needed to determine the relationship between general perceptual cognitive abilities and position groups.

### Practical applications

The results of this study suggest practical applications as follows: Practitioners (e.g. coaches) should consider the development of attention phases in younger age classes (U12-U15). Training contents with high and sustained attention requirements (e.g. tactical training) should have lower priority in these age classes. For example, these contents could be trained with ludic techniques and procedures transformed into a game. Due to enhanced attention skills for age classes starting from the U16 training contents with high attention demands could be implemented.

Our findings indicate that in younger age classes (U12-U15) reaction training could be very effective. Reaction training programs should be implemented with a perception-action approach in these age classes. Therefore, in practical use visual and acoustically perceived stimuli should be coupled directly with sport-specific reactions (e.g. dribbling, passing, running). These training contents could be transformed in playful forms.

Our position analysis indicates different position-specific behavioral patterns. We suggest that players with a great readiness to accept risks in general sustained attention testsmight be more successful as strikers, whereas players with less risk-taking could be rather successful in defence position. In addition, psychological support could be of advantage to develop strategies for a balance between risk-taking and safety behavior. Above-average reaction skills (visual and acoustic) likely give an indication if players might be successful in midfield. Improvements of soccer abilities of defenders and strikers could be achieved by a sport-specific reaction training.

## Conclusions

Summarizing we conclude that the present study delivers several findings referring to the impact of age on general perceptual-cognitive abilities and on the relation between playing position and abilities in highly talented soccer players. Our hypothesis that soccer position and age are related to general cognitive-perceptual abilities is confirmed by the following results: (1) We found correlations between age and CR. The differences in sustained attention (CR and E) between the age groups were significant. (2) The analysis of playing position revealed significant group differences in acoustic reaction time (ART) and statistical tendency for group differences in visual reaction time (VRT) and correct responses (CR). (3) Time and movement anticipation (TMA) measures demonstrate no group differences for age or position.

Above results might support the *Cognitive component skill approach* and clarify the need of future research dedicated to general perceptual-cognitive abilities in highly talented athletes. Also, the present findings offer indications, that figural sustained attention tasks are a strong age predictor for highly talented soccer players. Moreover, evidence was found for position-specific differences in visual and acoustic reaction tasks, indicating that midfielders outperform strikers and defenders in simple reaction times.

We suggest that future research is needed to clarify the development of general perceptual-cognitive abilities and position-specific differences in these abilities. Such studies should aim to investigate the relationship between soccer-specific skills and general attention and anticipation abilities using approaches which also integrate physiological parameters in order to better understand the underlying mechanisms.
